# Supplementary material for: NMR Structure Determinations of Small Proteins Using Only One Fractionally 20% 13C- and Uniformly 100% 15N-Labeled Sample
Source: Molecules. 2021 Feb 1;26(3):747. doi: 10.3390/molecules26030747 (PMC7867066; doi:10.3390/molecules26030747)
Supplement: Supplementary file 1 [file molecules-26-00747-s001.pdf]

molecules

MDPI

Supplemental Materials (Figure S1-S4)

NMR Structure Determinations of Small Proteins Using Only One Fractionally 20% ^{13}C- and Uniformly 100% ^{15}N-Labeled Sample

Harri. A. Heikkinen, Sofia M. Backlund and Hideo Iwaï *

Institute of Biotechnology, University of Helsinki. PO Box 65, Helsinki, FIN-00014, Finland; harri.a.heikkinen@helsinki.fi (H.A.H.); sofia.backlund@helsinki.fi (S.M.B.)

* Correspondence: hideo.iwai@helsinki.fi; Tel.: +358-2941-59752

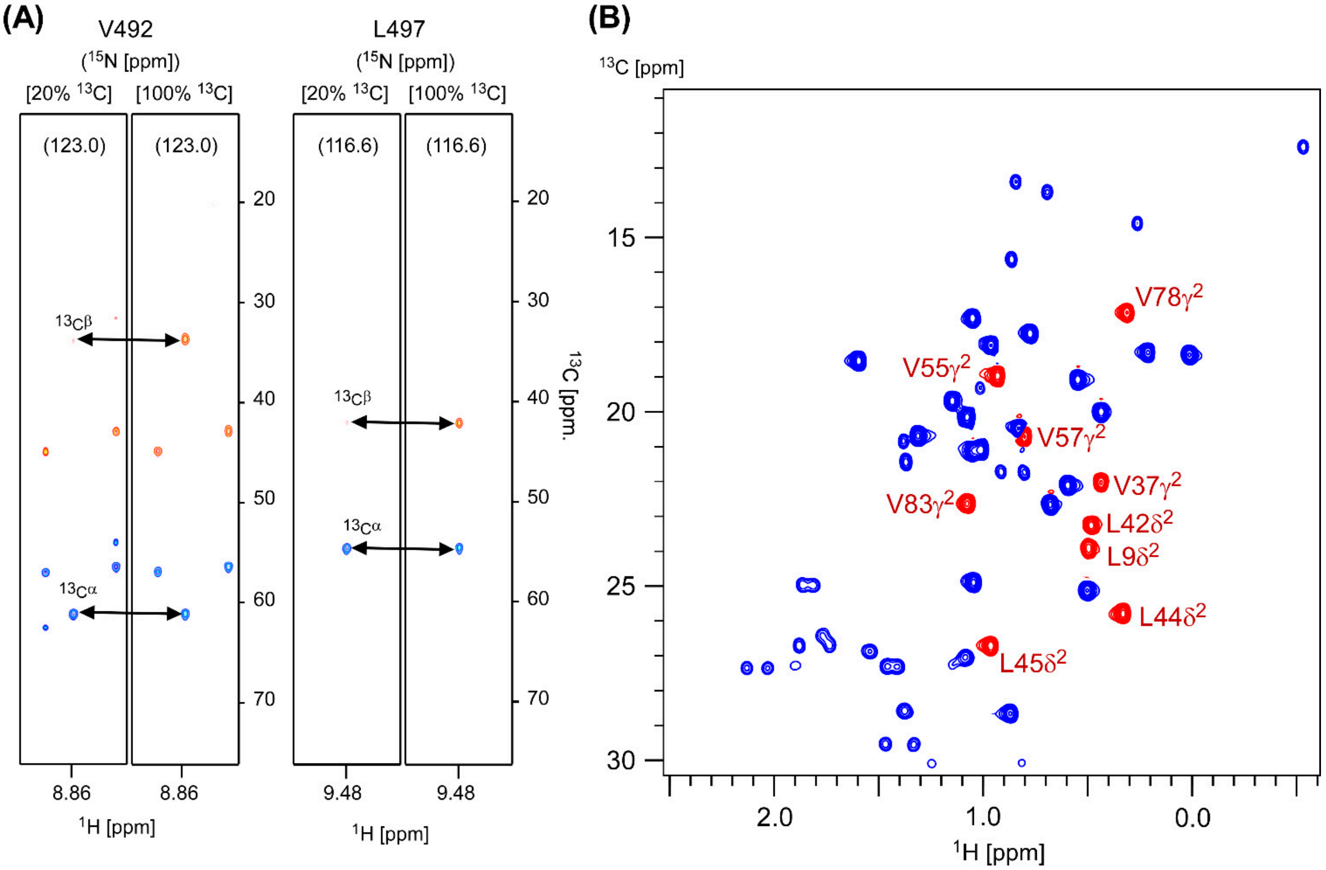

Supplemental Figure S1. (**A**) Comparison of intra-HNCACB spectra between [20% ^{13}C, 100% ^{15}N]-labeled and 100% [^{13}C, ^{15}N]-labeled samples. The fractional 20% ^{13}C-labeling scheme results in decreased signal intensities for ^{13}Cβ correlation peaks with respect to ^{13}Cα correlation peaks depending on the amino-acid types due to the breakages of $^{13}C^{\alpha}$-$^{13}C^{\beta}$ bonds during the amino-acid biosynthesis. Examples for the worst examples for residues V492 and L497 are shown by two strips from 3D intra-HNCACB spectra of [20% ^{13}C, 100% ^{15}N]-labeled sample (left) and 100% [^{13}C, ^{15}N]-labeled sample (right). Arrows indicate corresponding peaks. There are additional peaks in the fractional labeled samples (see the text). (**B**) ct-[^{1}H,^{13}C]-HSQC spectrum of [20% ^{13}C, 100% ^{15}N]-labeled CBM64. The constant evolution time was set to 1/Jcc so that *pro-S* and *pro-R* methyl groups have different signs in the spectra. The peaks for δ^2 methyl groups of Leu and γ^2 methyl groups of Val are shown in the negative (red) with the residue number.

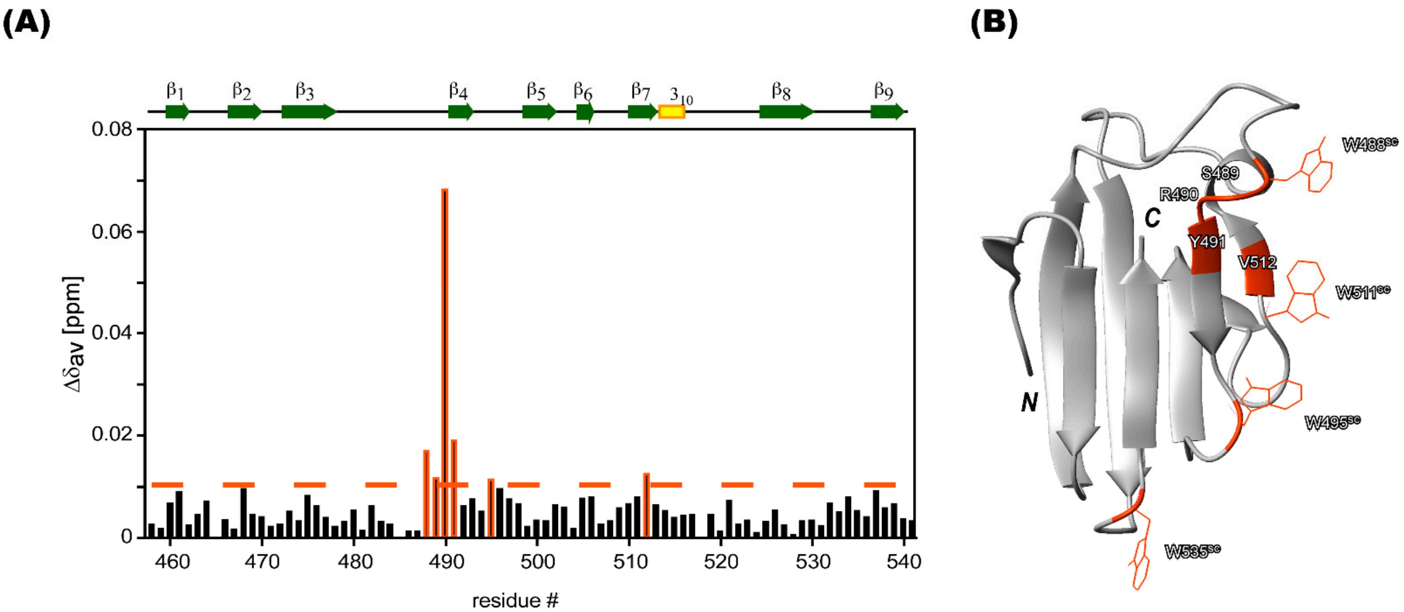

Supplemental Figure S2. Interaction analysis of CBM64 with cellobiose using CSP **(A)** Chemical shift perturbation (CSP) upon the addition of D-cellobiose. CSP was calculated using the equation $\Delta\delta_{av} = [(\delta H^N)^2 + (0.154 \times \delta N^H)^2]^{1/2}$. The dashed line indicates the threshold value ($\Delta\delta_{av} > 0.01$ ppm) used as the criteria for mapping residues on the protein. The secondary structures are shown above the plots. **(B)** Chemical shift perturbations mapped on the CBM64 structure in orange, where backbone amides and indole-amine groups above threshold CSP value (> 0.01 ppm) are highlighted.

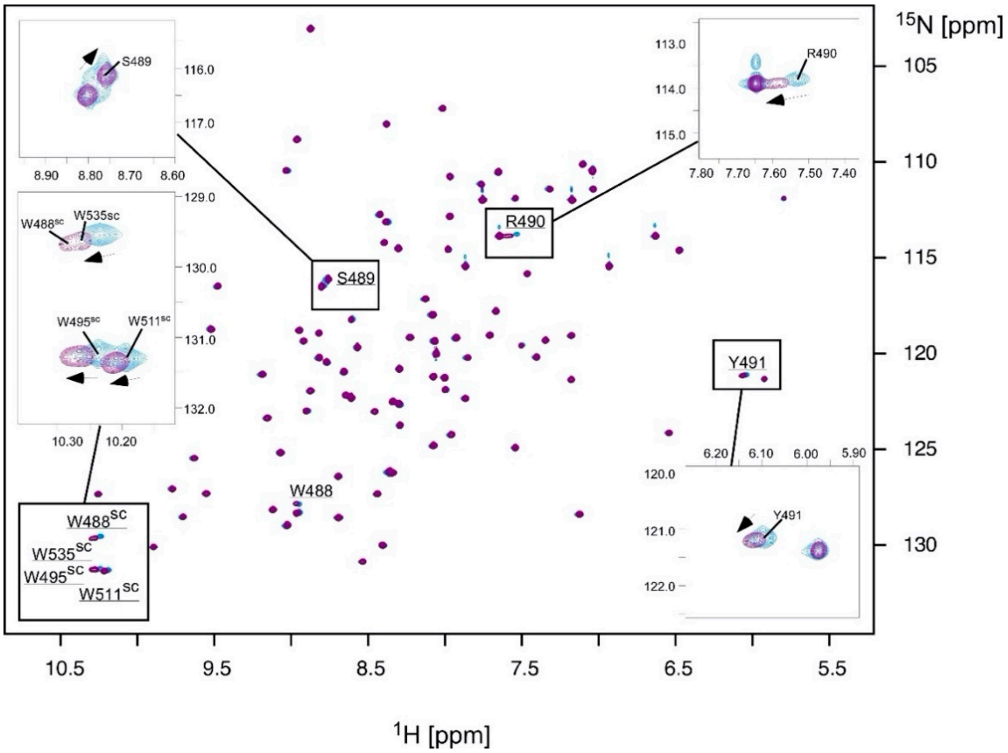

Supplemental Figure S3. Titration of CBM64 by addition of cellobiose using HSQC spectra. An overlay of the [^{1}H, ^{15}N]-HSQC NMR spectra of 0.25 mM CBM64 showing chemical shift changes upon the addition of 6.25 mM D-cellobiose recorded at the ^{1}H frequency of 850 MHz, 303 K. The spectra with and without D-cellobiose were colored in violet and blue, respectively. The amide residues (W488-Y491) and indole-amine groups (W488^{sc} (CSP 0.07 ppm), W495^{sc} (CSP 0.03 ppm),

W511[sc] (CSP 0.03 ppm), and W535[sc] (CSP 0.05 ppm) displaying the highest CSPs (> 0.01 ppm) are highlighted.

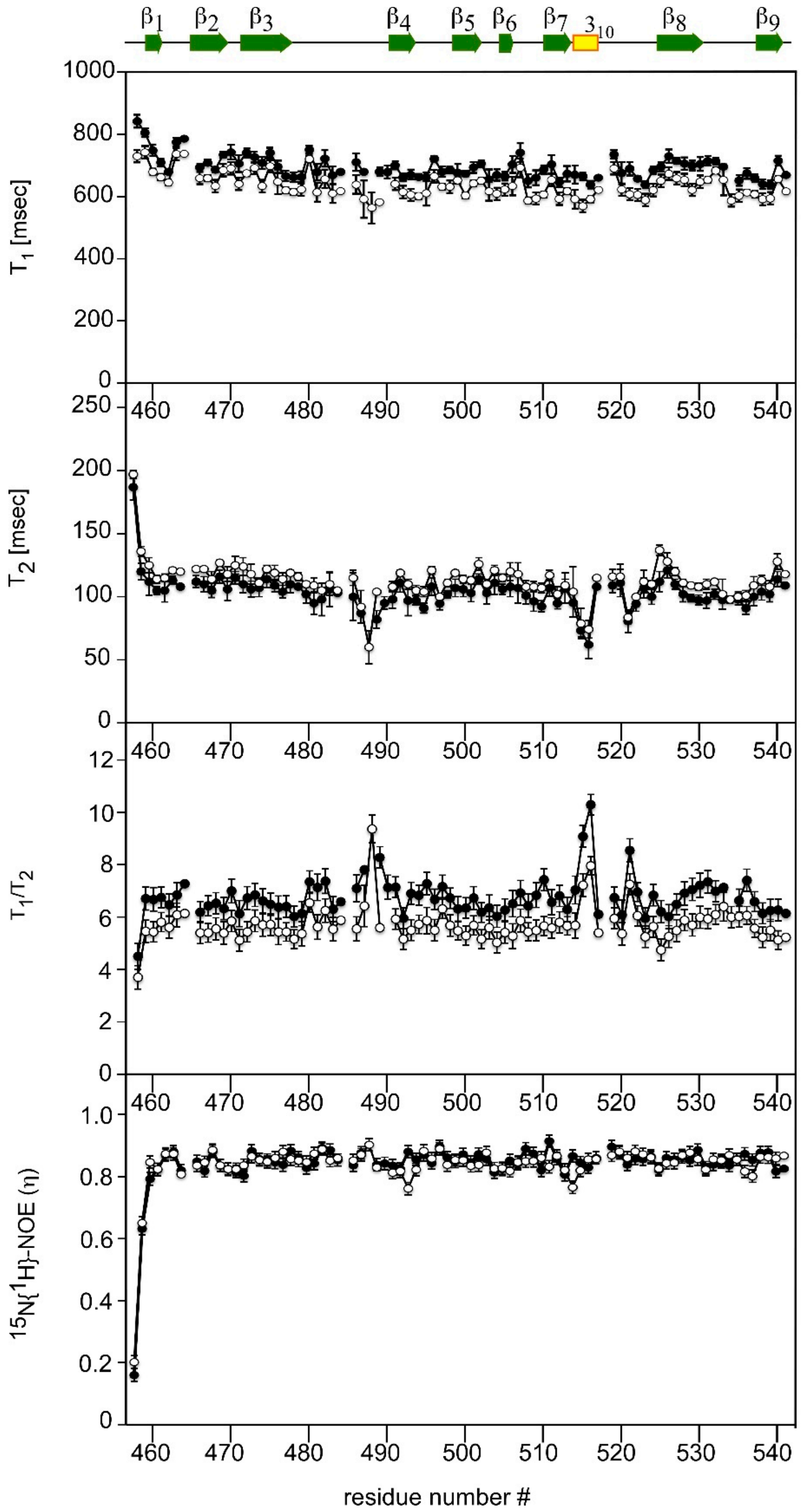

Supplemental Figure S4. ^{15}N relaxation analysis with and without cellobiose. ^{15}N relaxation data obtained with 0.25 mM [20% ^{13}C, 100% ^{15}N]-labeled CBM64 sample without ligand (filled circles) and in the presence of 6.25 mM D-cellobiose (open circles). Longitudinal relaxation times (T_1), transverse relaxation times (T_2), and $^{15}N\{^1H\}$-NOE values were recorded at the ^1H frequency of 850 MHz and 303 K and plotted together with T_1/T_2 ratios. Secondary structure elements are shown above the plots.
